# NETs Promote Inflammatory Injury by Activating *cGAS-STING* Pathway in Acute Lung Injury

**DOI:** 10.3390/ijms24065125

**Published:** 2023-03-07

**Authors:** Jie Zhao, Ningxin Zhen, Qichao Zhou, Jian Lou, Wei Cui, Gensheng Zhang, Baoping Tian

**Affiliations:** 1 Department of Critical Care Medicine, The Second Affiliated Hospital, Zhejiang University School of Medicine, 88 Jiefang Rd., Hangzhou 310009, China; 2 Department of Respiratory and Critical Care Medicine, The Second Affiliated Hospital, Zhejiang University School of Medicine, Hangzhou 310009, China

**Keywords:** acute respiratory distress syndrome, acute lung injury, neutrophil extracellular traps, inflammatory injury, *cGAS-STING* pathway

## Abstract

Acute respiratory distress syndrome (ARDS) threatens the survival of critically ill patients, the mechanisms of which are still unclear. Neutrophil extracellular traps (NETs) released by activated neutrophils play a critical role in inflammatory injury. We investigated the role of NETs and the underlying mechanism involved in acute lung injury (ALI). We found a higher expression of NETs and cyclic GMP-AMP synthase-stimulator of interferon genes (*cGAS-STING*) in the airways, which was reduced by Deoxyribonuclease I (DNase I) in ALI. The administration of the *STING* inhibitor H-151 also significantly relieved inflammatory lung injury, but failed to affect the high expression of NETs in ALI. We isolated *murine* neutrophils from bone marrow and acquired *human* neutrophils by inducing HL-60 to differentiate. After the PMA interventions, exogenous NETs were obtained from such extracted neutrophils. Exogenous NETs intervention in vitro and in vivo resulted in airway injury, and such inflammatory lung injury was reversed upon degrading NETs with or inhibiting *cGAS-STING* with H-151 as well as siRNA *STING*. In conclusion, *cGAS-STING* participates in regulating NETs-mediated inflammatory pulmonary injury, which is expected to be a new therapeutic target for ARDS/ALI.

## 1. Introduction

Acute respiratory distress syndrome (ARDS) is an acute, diffuse inflammatory lung injury caused by multiple intra-pulmonary and extra-pulmonary factors [1]. Pneumonia, aspiration of gastric contents, pulmonary contusion, sepsis, trauma, burns, blood product transfusion and coronavirus disease 2019 (COVID-19) are common risk factors of ARDS [1,2]. A large study involving 459 intensive care units (ICUs) from 50 countries reported that 10% of ICU patients and 23% of patients with mechanical ventilation fulfilled criteria for ARDS [3]. Hospital mortality for ARDS was consistently reported to be greater than 30% in observational studies, and 90-day in-hospital mortality even reached 43% for moderate to severe ARDS [4]. Although numerous investigations were performed, regrettably, there is no significant progress in the diagnosis or treatment of ARDS [5]. The major strategies are symptomatic and supportive treatment, including ventilator support, prone position, fluid management and glucocorticoids. No effective drugs have been found over the decades of investigating pharmacotherapies for ARDS [2,6,7,8,9].

Infiltration of neutrophils in the lung is an important pathological manifestation of ARDS. Neutrophils from patients with ARDS are activated, which have enhanced chemotaxis, enhanced metabolic activity, delayed apoptosis and a novel transcriptional signature [10,11]. Neutrophil extracellular traps (NETs) are released by activated neutrophils and are reticular complexes composed of double-stranded DNA (dsDNA) and various proteins such as myeloperoxidase (MPO), neutrophil elastase (NE) and histone [12]. NETs are two-faced: on the one hand, they can immobilize and disarm invaders; on the other hand, over-production of NETs is correlated with cytotoxic, pro-inflammatory and pro-thrombotic events. Acute lung injury (ALI) is mild ARDS, and animal models of ALI are commonly utilized to perform associated investigations of ARDS. Recent studies showed the biological relevance of NETs in the pathogenesis of acid-aspiration- or Lipopolysaccharide (LPS)-induced ALI, transfusion-related ALI and COVID-19-related ARDS [13,14,15,16]. Large numbers of infiltrating neutrophils produce NETs, and NETs over-accumulate in the pulmonary tissue and alveolar space owing to the imbalance of production and degradation in pathological conditions. Currently, the specific mechanism of NETs involved in the pathogenesis of ARDS/ALI is still unclear, which is a significant scientific issue deserving further exploration. In the present study, we aimed to investigate the role of NETs in the inflammatory injury process of ALI and elucidate its specific mechanism, favoring the exploration of new biological markers and therapeutic targets of ARDS/ALI.

During the initial exploration of the mechanism, we found that cytosolic DNA sensing pathways were over-activated in the lung of an LPS-induced ALI *murine* model. *Cyclic GMP-AMP synthase* (*cGAS*) is a cytosolic DNA sensor, which converts ATP and GTP into cyclic GMP-AMP (cGAMP) when binding with self-dsDNA or extracellular dsDNA. Stimulator of interferon genes (*STING*) activated by cGAMP translocates from the endoplasmic reticulum (ER) to the Golgi apparatus, and is then recruited and phosphorylated by TANK-binding kinase 1 (TBK1) and interferon regulatory factor 3 (IRF3), eventually inducing type I interferon (IFN-I)- and NF-kB-mediated inflammatory responses [17]. Moreover, the activation of the *cGAS-STING* pathway also triggers various cell death pathways such as autophagy, pyroptosis, necrosis and apoptosis [18]. Recently, evidence showed that the *cGAS-STING* pathway over-activation is involved in multiple inflammatory lung diseases including cystic fibrosis (CF), chronic obstructive pulmonary disease (COPD), idiopathic pulmonary fibrosis (IPF), asthma and COVID-19 [19,20]. A previous study showed that NETs engulfed by macrophages and other myeloid cells translocated to the cytosol, where the DNA backbone of NETs activated the *cGAS-STING* pathway and induced IFN-I production [21]. Thus, the dsDNA content in NETs might be a potent stimulator of the *cGAS-STING* pathway and induce an inflammatory response in addition to cellular damage in ARDS/ALI.

In summary, we conjecture that NETs might be involved in the regulation of inflammatory injury through the *cGAS-STING* pathway in acute lung injury. We intended to investigate the specific mechanism of the *cGAS-STING* pathway in the pathological process of NETs-mediated ALI, further elucidating the pathophysiological mechanism of ARDS and providing a theoretical basis for exploring new biological markers and treatment strategies.

## 2. Results

### 2.1. NETs Are Increased in Mice with LPS-Induced Acute Lung Injury

**Figure 1:**
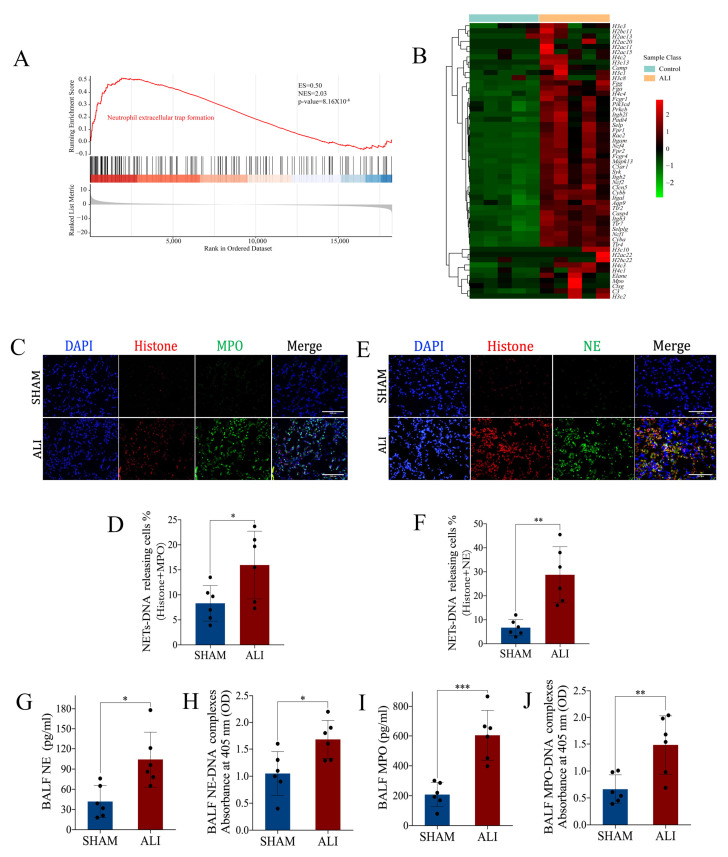
NETs are increased in *mice* with LPS-induced acute lung injury. Lungs from the ALI model and control were obtained to perform RNA-seq analysis. (**A**) Enrichment plot of the neutrophil extracellular trap formation signaling pathway from GSEA comparing ALI vs. control. (**B**) Heatmap showing the core genes enriched in GSEA in the neutrophil extracellular trap formation signaling pathway. The abscissa indicates the sample number, the ordinate indicates the differentially expressed genes, the right histogram indicates the color scale and each rectangle in the panel corresponds to an expression value in one sample. (**C**) Representative images of immunofluorescence co-localization of histone and MPO (scale bar = 200 µm). (**D**) The percentage of NET-DNA (histone + MPO)-releasing cells. (**E**) Representative images of immunofluorescence co-localization of histone and NE (scale bar = 200 µm). (**F**) The percentage of NET-DNA (histone + NE)-releasing cells. (**G**) NE, (**H**) NE-DNA, (**I**) MPO and (**J**) MPO-DNA concentrations in BALF. GSEA = gene set enrichment analysis; ES = enrichment score; NES = normalized enrichment score; ALI = acute lung injury. Data are presented as the mean ± SD, n = 6 per group, * *p* < 0.05, ** *p* < 0.01, *** *p* < 0.001. Comparisons between two groups were analyzed by an unpaired *t* test.

We validated the ALI *murine* model in our laboratory via intratracheal instillation of LPS (10 mg/kg). The ALI model induced by LPS for 24h had apparent pathological manifestations of lung injury such as pulmonary edema, pulmonary hemorrhage, thickening of the alveolar septum and inflammatory cell infiltration (Supplementary Figure S1A,B); increased lung wet weight-to-dry weight ratio (Supplementary Figure S1C); and increased pulmonary vascular permeability (Supplementary Figure S1D). γH2AX was a marker indicating DNA damage. The immunofluorescence staining results showed an elevated percentage of γH2AX-positive SFTPC+ cells in pulmonary tissue of the ALI *murine* model, suggesting LPS-induced DNA damage in type II alveolar epithelial cells (AT-II) (Supplementary Figure S1E). These results demonstrated the successful establishment of the ALI *murine* model. We performed a transcriptomic analysis on the sequencing data of the lung from the ALI *murine* model and control. The Kyoto Encyclopedia of Genes and Genomes (KEGG) enrichment plots obtained from Gene Set Enrichment Analysis (GSEA) showed that the majority of the neutrophil extracellular trap formation pathway-associated genes were up-regulated in ALI (Figure 1A,B). To identify the expression of NETs in this LPS-induced ALI *murine* model, the quantification of NETs in bronchoalveolar lavage fluid (BALF) was examined by ELISA, and the quantification of NETs in the lung was carried out with immunofluorescence staining. MPO, NE, histone and DNA are known biomarkers for identifying NETs. Compared with the control, the expression of NETs in the lung (Figure 1C–F) and BALF (Figure 1G–J) increased significantly in the ALI model. Taken together, these results indicated that NETs increased in LPS-induced acute lung injury.

### 2.2. Pharmacological Degradation of NETs Confers Protection against LPS-Induced Acute Lung Injury

**Figure 2:**
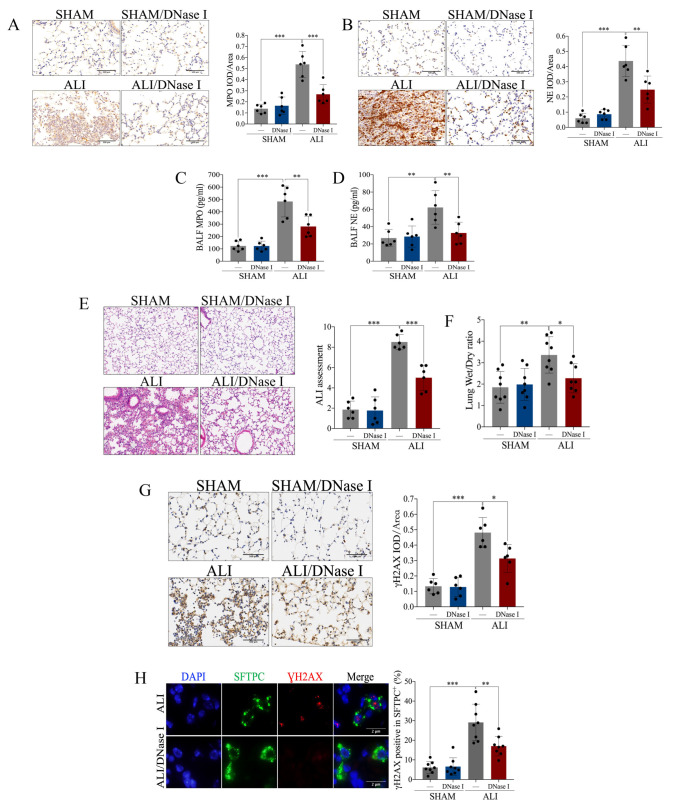
Pharmacological degradation of NETs confers protection against LPS-induced acute lung injury. NET inhibitor DNase I (20 mg/kg) was intravenously injected 1 h after LPS treatment. (**A**) Representative images of the immunohistochemical staining of MPO (scale bar = 500 µm), and statistics of the positive signal of MPO in lung tissue. (**B**) Representative images of immunohistochemical staining of NE (scale bar = 500 µm), and statistics of the positive signal of NE in lung tissue. (**C**) Concentration of MPO in BALF. (**D**) Concentration of NE in BALF. (**E**) Representative images of H&E staining of lung tissue (X100), and ALI assessment score. (**F**) Lung wet weight-to-dry weight ratio. (**G**) Representative images of immunohistochemical staining of γH2AX in lung tissue (scale bar = 500 µm), and statistics of the positive signal of γH2AX in lung tissue. (**H**) Representative images of immunofluorescence co-localization of SFTPC and γH2AX (scale bar = 2 µm), and the percentage of γH2AX-positive SFTPC+ cells. Data are presented as the mean ± SD, n = 6–8 per group. * *p* < 0.05, ** *p* < 0.01, *** *p* < 0.001. Comparisons between two groups were analyzed by an unpaired t test; otherwise, one-way analysis of variance was performed, and Tukey’s test was used to perform pairwise comparisons.

Having identified an elevation in NETs in the LPS-induced ALI *murine* model, we continued to explore whether NETs participate in the pathogenesis of ALI. Deoxyribonuclease I (DNase I), which can pharmacologically degrade NETs, and which was intravenously injected into the ALI *murine* model and control. The expression of MPO and NE was elevated in the lung and BALF of the ALI model, which was diminished by the treatment with DNase I (Figure 2A–D). The above results indicated that DNase I successfully degraded NETs induced by LPS. Pathological manifestations including pulmonary edema, hemorrhage, thickening of the alveolar septum and infiltration of inflammatory cells (Figure 2E) were displayed in the LPS-induced *murine* model, accompanied by an increased lung wet weight-to-dry weight ratio (Figure 2F), and elevated DNA damage in pulmonary tissue and AT-II (Figure 2G, H). Interestingly, the above changes in the ALI *murine* model were reversed in response to the pharmacological degradation of the NETs by DNase I (Figure 2E–H). In brief, NETs participated in the pathophysiological process of LPS-induced acute lung injury, and pharmacological degradation of NETs conferred protection against ALI.

### 2.3. cGAS-STING Is Up-Regulated in LPS-Induced Acute Lung Injury

**Figure 3:**
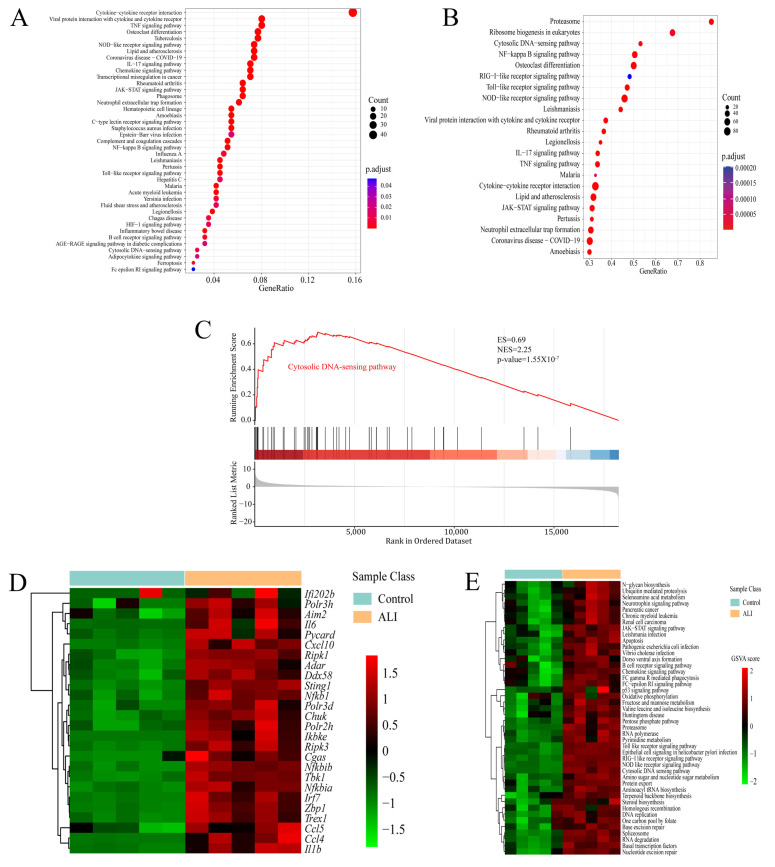
RNA-seq analysis suggests that the cytosolic DNA sensing pathway is up-regulated in acute lung injury. Lungs from the ALI model and control were obtained to perform RNA-seq analysis. (**A**) Bubble chart of KEGG enrichment analysis for significantly up-regulated genes in ALI (log2 foldchange > 2 and adjust *p* < 0.01). (**B**) Bubble chart of KEGG enrichment in GSEA showing up-regulated pathways in ALI (NES > 2 and *p*-value < 0.05). The ordinate indicates the terms of the enriched pathways, the abscissa indicates the GeneRatio and the size of the bubble corresponds to the count of genes in each term. The color intensity of the bubble indicates adjust *p*, while the intensity follows the color bar on the right. (**C**) Enrichment plot of the cytosolic DNA sensing pathway from GSEA comparing ALI vs. control. (**D**) Heatmap showing the core genes enriched in GSEA in the cytosolic DNA sensing pathway. The abscissa indicates the sample number, the ordinate indicates the differentially expressed genes, the right histogram indicates the color scale and each rectangle in the panel corresponds to the expression value in one sample. (**E**) Heatmap showing KEGG pathways up-regulated in ALI based on GSVA. The abscissa indicates the sample number, the ordinate indicates the KEGG pathway term, the right histogram indicates the color scale and each rectangle in the panel corresponds to the GSVA score. ALI = acute lung injury; KEGG = Kyoto Encyclopedia of Genes and Genomes; GSEA = gene set enrichment analysis; GSVA = gene set variation analysis; NES = normalized enrichment score.

**Figure 4:**
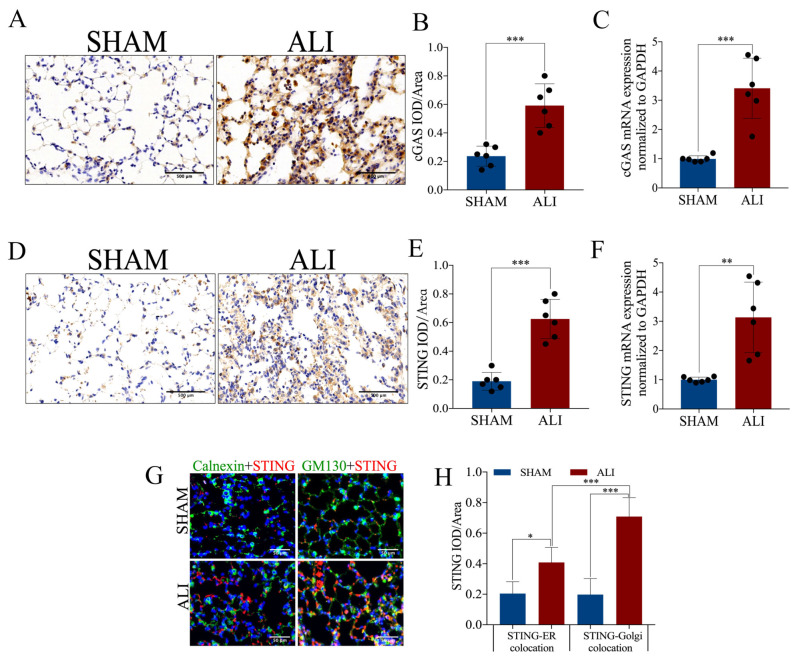
*cGAS-STING* expression is up-regulated in LPS-induced acute lung injury. (**A**) Representative images of immunohistochemical staining of *cGAS* (scale bar = 500 µm). (**B**) Statistics of the positive signal of *cGAS* in lung tissue. (**C**) *cGAS* mRNA expression in lung tissue. (**D**) Representative images of immunohistochemical staining of *STING* (scale bar = 500 µm). (**E**) Statistics of the positive signal of *STING* in lung tissue. (**F**) *STING* mRNA expression in lung tissue. (**G**) Representative images of immunofluorescence co-localization of calnexin (green) and *STING* (red), and GM130 (green) and *STING* (red) (scale bar = 50µm). (**H**) Fluorescence signal statistics of *cGAS* and *STING* located in the ER and Golgi. IOD = integral optical density. Data are presented as the mean ± SD, n = 6 per group. * *p* < 0.05, ** *p* < 0.01, *** *p* < 0.001. Comparisons between two groups were analyzed by an unpaired *t* test.

A total of 629 genes up-regulated in ALI (log2 foldchange > 2 and adjust *p* < 0.01) were included, and KEGG enrichment analysis of such genes revealed that neutrophil extracellular trap formation, the cytosolic DNA sensing pathway and its related pathways such as the NF-kappa B signaling pathway, cytokine–cytokine receptor interaction and the chemokine signaling pathway were enriched in the up-regulated differentially expressed genes (DEGs) in ALI (Figure 3A). In addition to the conventional KEGG enrichment analysis, we also performed GSEA to observe the overall change trend of differential genes located on a certain pathway. The bubble chart of enriched pathways (normalized enrichment score, NES > 2) showed that neutrophil extracellular trap formation, the cytosolic DNA sensing pathway and its related pathways including the NF-kappa B signaling pathway and cytokine–cytokine receptor interaction were up-regulated in ALI (Figure 3B). The KEGG enrichment plots obtained by GSEA showed that the majority of the cytosolic DNA sensing pathway-associated genes were up-regulated in ALI (Figure 3C,D). Gene set variation analysis (GSVA) is a method that calculates the GSVA score of each gene set and carries out differential analysis of gene sets. The heatmap showing the pathways with a higher GSVA score in ALI (log2 foldchange > 0 and adjust *p* <0.05) revealed that the cytosolic DNA sensing pathway and its related pathways such as ubiquitin-mediated proteolysis, apoptosis, the chemokine signaling pathway and the RIG-I like receptor signaling pathway were up-regulated in ALI (Figure 3E). In brief, the bioinformatics analysis of RNA sequencing suggested that the cytosolic DNA sensing pathway, also named the *cGAS-STING* pathway, was activated in ALI. Then, we performed qRT-PCR and immunohistochemical staining to quantify the mRNA and protein expression of *cGAS* and *STING* in pulmonary tissue. Notably, both the mRNA and protein expressions of *cGAS* and STING were increased significantly in the ALI *murine* model compared to the control (Figure 4A–F). Calnexin is a marker of the ER, while GM130 is a marker of the Golgi apparatus. We performed immunofluorescence co-localization of *STING* with GM130 and Calnexin to further quantify the expression of *STING* located in the ER and Golgi, respectively. The immunofluorescence of *STING* + Calnexin and *STING* + GM130 showed increased expression of *STING* located in the ER and Golgi in the ALI *murine* model. Furthermore, the elevation in *STING* located in the Golgi was significantly higher than that of *STING* located in the ER, indicating that *STING* translocated from the ER to the Golgi, and that *cGAS-STING* was activated (Figure 4G,H). Taken together, the *cGAS-STING* pathway was activated in acute lung injury.

### 2.4. Pharmacological Blockade of cGAS-STING Confers Protection against LPS-Induced Acute Lung Injury

**Figure 5:**
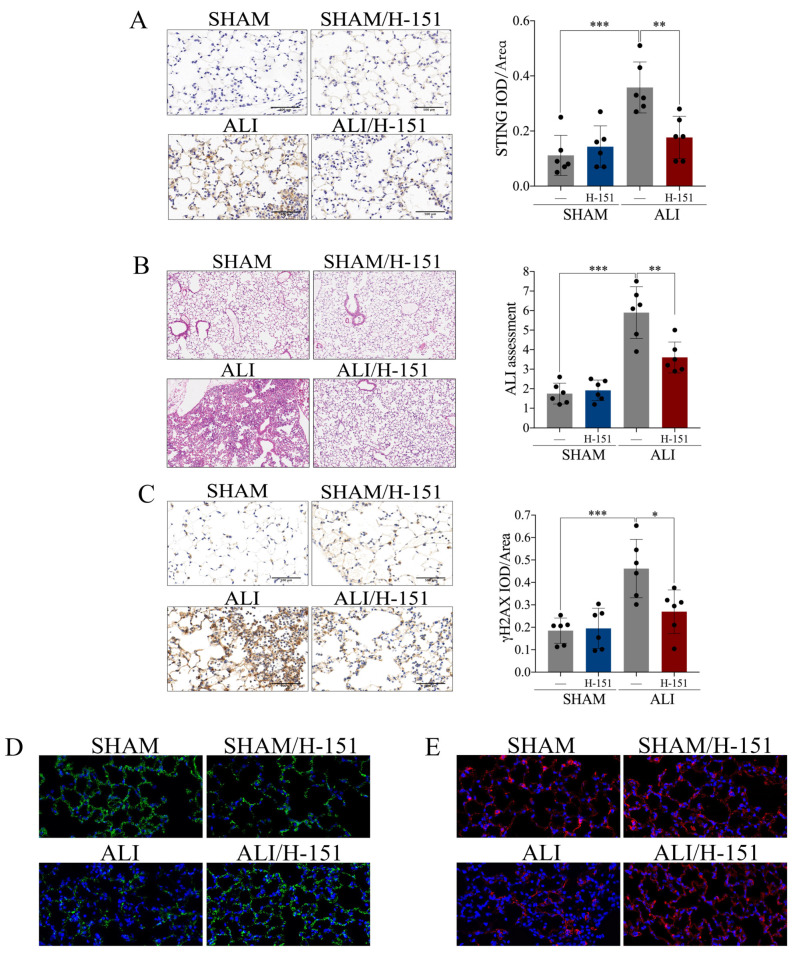
Pharmacological blockade of *cGAS-STING* confers protection against LPS-induced acute lung injury. *STING* inhibitor H-151 (30 mg/kg) was intraperitoneally injected 30 min before LPS administration. (**A**) Representative images of immunohistochemical staining of *STING* in lung tissue (scale bar = 500 µm), and statistics of the positive signal of *STING* in lung tissue. (**B**) Representative images of H&E staining of lung tissue (X100), and ALI assessment score. (**C**) Representative images of immunohistochemical staining of γH2AX in lung tissue (scale bar = 500 µm), and statistics of the positive signal of γH2AX in lung tissue. (**D**) Representative images of immunofluorescence staining of ZO-1 (green) in lung tissue (X400). (**E**) Representative images of immunofluorescence staining of E-cadherin (red) in lung tissue (X400). IOD = integral optical density. Data are presented as the mean ± SD, n = 6 per group, * *p* < 0.05, ** *p* < 0.01, *** *p* < 0.001. Comparisons between two groups were analyzed by an unpaired t test; otherwise, one-way analysis of variance was performed, and Tukey’s test was used to perform pairwise comparisons.

Having identified activation of the *cGAS-STING* pathway in LPS-induced ALI, we continued to explore whether *cGAS-STING* participates in the pathogenesis of ALI. H-151, an inhibitor of *STING*, was intraperitoneally injected into the ALI *murine* model and control. The increased expression of *STING* in the lung from the *murine* ALI model was reversed by the treatment with H-151, which indicated a successful blockade of the *cGAS-STING* pathway (Figure 5A). ZO-1 and E-cadherin are markers of intercellular junctions. Administration of H-151 alleviated pulmonary edema, hemorrhage, thickening of the alveolar septum and infiltration of inflammatory cells (Figure 5B); DNA damage in pulmonary tissue (Figure 5C); and disruption of intercellular junctions (Figure 5D,E) in LPS-induced ALI. Taken together, activation of *cGAS-STING* participated in the pathophysiological process of LPS-induced acute lung injury, and pharmacological blockade of *cGAS-STING* conferred protection against ALI.

### 2.5. NETs Induced Acute Lung Injury via cGAS-STING Pathway

**Figure 6:**
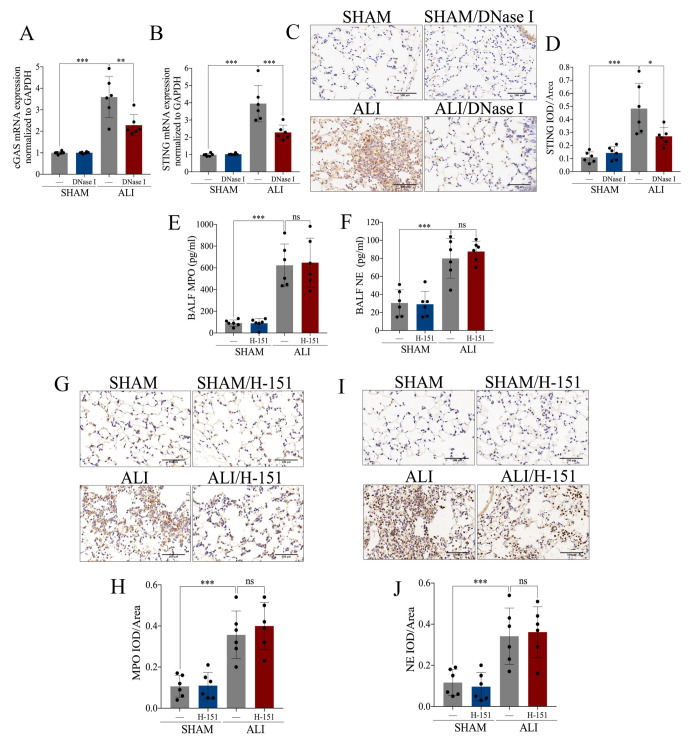
NETs induce acute lung injury via the *cGAS-STING* pathway. NETs inhibitor DNase I (20 mg/kg) was intravenously injected to degrade NETs. (**A**) mRNA expression of *cGAS* in lung tissue. (**B**) mRNA expression of *STING* in lung tissue. (**C**) Representative images of immunohistochemical staining of *STING* in lung tissue (Scale bar = 500 µm). (**D**) Statistics of the positive signal of *STING* in lung tissue. *STING* inhibitor H-151 (30 mg/kg) was intraperitoneally injected as a *cGAS-STING* blockade. (**E**) Concentration of MPO in BALF. (**F**) Concentration of NE in BALF. (**G**) Representative images of immunohistochemical staining of MPO in lung tissue (Scale bar = 500 µm). (**H**) Statistics of the positive signal of MPO in lung tissue. (**I**) Representative images of immunohistochemical staining of NE in lung tissue (Scale bar = 500 µm). (**J**) Statistics of the positive signal of NE in lung tissue. IOD = integral optical density; ns = no significance. Data are presented as the mean ± SD, n = 6 per group, * *p* < 0.05, ** *p* < 0.01, *** *p* < 0.001. Comparisons between two groups were analyzed by an unpaired t test; otherwise, one-way analysis of variance was performed, and Tukey’s test was used to perform pairwise comparisons.

We identified that both NETs and *cGAS-STING* were involved in the pathophysiological process of LPS-induced ALI. The mechanism of whether a relationship between NETs and *cGAS-STING* exists or not attracted our attention. On the one hand, the mRNA expression of *cGAS* as well as *STING* (Figure 6A,B) and the protein expression of *STING* (Figure 6C,D) were increased in pulmonary tissue of the ALI *murine* model. Interestingly, the above changes were reversed when NETs were degraded effectively by DNase I (Figure 6A–D). On the other hand, MPO and NE in BALF (Figure 6E,F) and pulmonary tissue (Figure 6G–J) were increased in the ALI *murine* model. Furthermore, there was no significant expressional decline in NETs when blockade of *cGAS-STING* with H-151 was applied (Figure 6E–J). In summary, the degradation of NETs inhibited *cGAS-STING*, while blockade of *cGAS-STING* did not affect the increased expression of NETs in ALI. We concluded that NETs participated in the pathological process of acute lung injury via the *cGAS-STING* pathway.

### 2.6. Exogenous NETs Induce Epithelial Cell Damage via cGAS-STING In Vitro and In Vivo

**Figure 7:**
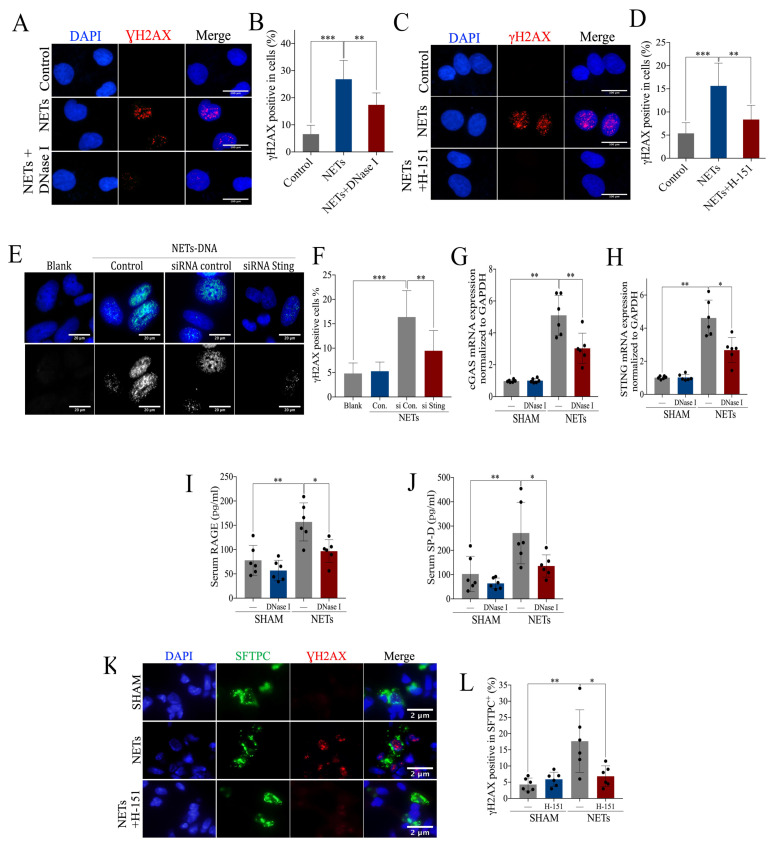
Intervention of exogenous NETs induces airway injury via *cGAS-STING*. (**A**) Representative images of immunofluorescence staining of γH2AX in BEAS-2B cells pre-incubated with exogenous NETs and DNase I (Scale bar = 100 µm*),* and (**B**) statistics of the γH2AX-positive cells. (**C**) Representative images of immunofluorescence staining of γH2AX in BEAS-2B cells pre-incubated with exogenous NETs and H-151 (Scale bar = 100 µm), and (**D**) statistics of the γH2AX-positive cells. (**E**) Representative images of immunofluorescence staining of γH2AX in STING siRNA BEAS-2B cells pre-incubated with NETs (Scale bar = 20 µm), and (**F**) statistics of the γH2AX-positive cells. mRNA expression of (**G**) *cGAS* and (**H**) *STING* in lung tissue of *mice* administered with exogenous NETs and DNase I. Concentration of serum (**I**) RAGE and (**J**) SP-D, for *mice* administered with exogenous NETs and DNase I. (**K**) Representative images of immunofluorescence co-localization of γH2AX and SFTPC (Scale bar = 2 µm) and (**L**) the percentage of γH2AX-positive SFTPC+ cells. Data are presented as the mean ± SD, n = 6 per group, * *p* < 0.05, ** *p* < 0.01, *** *p* < 0.001. Comparisons between two groups were analyzed by an unpaired t test; otherwise, one-way analysis of variance was performed, and Tukey’s test was used to perform pairwise comparisons.

To further verify the effects of NETs on pulmonary epithelial cells, we first stimulated the bronchial epithelium transformed with Ad12-SV40 2B (BEAS-2B) with exogenous *human*-derived NETs (hNETs), and found that the percentage of γH2AX-positive BEAS-2B cells increased after stimulation with hNETs (Figure 7A–F). Moreover, we also found that DNA damage was enhanced in BEAS-2B cells after stimulation with hNETs, which was reversed in response to co-incubation with DNase I, further suggesting that hNETs induce DNA damage in pulmonary epithelial cells (Figure 7A,B). Furthermore, DNA damage in BEAS-2B cells induced by hNETs decreased significantly when BEAS-2B cells were co-incubated with STING inhibitor H-151 (Figure 7C,D) or *STING* was knocked down by small interfering RNA (Figure 7E,F), indicating hNETs induced pulmonary epithelial cellular DNA damage via *cGAS-STING* in vitro. Elevated mRNA expression of *cGAS* and *STING* was observed in *mice* receiving *murine*-derived NETs (mNETs) by intratracheal instillation, which was diminished by the administration of DNase I (Figure 7G,H), suggesting mNETs induced activation of *cGAS-STING* in vivo. Receptor of advanced glycation end products (RAGE) and surfactant protein-D (SP-D) are markers of airway injury [22,23,24]. RAGE as well as SP-D (Figure 7I–J) and the percentage of γH2AX-positive SFTPC+ cells (Figure 7K,L) were increased in *mice* with exogenous mNETs, suggesting NETs induced epithelial cellular damage in vivo. Additionally, the blockade of *cGAS-STING* significantly alleviated the mNETs-induced DNA damage, as shown by the decreased percentage of γH2AX-positive SFTPC+ cells when the ALI model was treated with H-151 (Figure 7K,L). In summary, the hNETs intervention experiments in vitro and mNETs intervention experiments in vivo showed that NETs induce pulmonary epithelial cell damage via *cGAS-STING*.

## 3. Discussion

ARDS is still a life-threatening disease with high morbidity and mortality in critically ill patients, for whom early diagnosis and effective therapy are important to experience better outcomes [1,2]. However, the etiology of ARDS is complicated and the underlying mechanism has not yet been determined. We observed over-production of NETs-related components and over-activation of *cGAS-STING* in LPS-induced ALI. In this study, we demonstrated that NETs were involved in the pathogenesis of ALI. Notably, we first found that the *cGAS-STING* pathway also participated in the pathological process of acute lung injury, and that *cGAS-STING* regulated NETs-mediated inflammatory pulmonary injury, which is full of novelty and clinical value.

NETs are released by activated neutrophils and play a critical role in host defense and various *human* diseases [25]. NETs are complexes of multiple components such as DNA, MPO, NE and histones. DNase I is capable of digesting NETs-DNA scaffolds. Liu et al. demonstrated that DNase I promoted NETs-protein and protected *mice* from acute lung injury [14]. In our study, administration of DNase I not only reversed the elevated expression of other NETs-related components such as NE and MPO in ALI, but also relieved LPS-induced inflammatory pulmonary injury. We found that diffuse alveolar injury is severe in ALI, and that the vascular leakage is remarkable. In brief, DNase I might have some therapeutic effects for acute lung injury, which is consistent with the findings of previous studies performed by Li et al. and Czaikoski et al. [26,27]. Czaikoski et al. revealed that DNase I relieved tissue injury and increased the survival rate of *mice* [27]. In our study, *mice* received intravenous administration of DNase I, in a way that not only delivered sufficient doses of DNase I to pulmonary tissue via the circulatory system, but also avoided aggravating pulmonary injury by secondary airway administration. However, intravenous administration of DNase I also degraded circulating NETs and impacted the internal environment; thus, the mitigating effects of DNase I on acute lung injury might be the result of a combination of more factors. Pulmozyme is a recombinant *human* alfa deoxyribonuclease I (rh-DNase I), which is used to treat patients with pulmonary fibrosis by degrading DNA in sputum. Recent studies that focused on patients with ARDS secondary to COVID-19 found that aerosolized pulmozyme had positive effects in relieving acute respiratory failure and improving patients’ outcomes [28,29]. However, rh-DNase I was metabolized rapidly, whereas the pulmonary inflammation was persistent, which made it difficult for therapeutic effects to be maintained for a long time [29,30]. Thus, novel therapies targeting NETs-related DNA might be a promising treatment. More relevant basic and clinical studies need to be carried out to clarify the effects and optimize current treatment strategies. Another NETs-related component, NE, is believed to be an important mediator involved in ALI; however, no clinical studies supported the idea that administration of sivelestat (a selective inhibitor of NE) has benefits on improving the outcomes of patients with ARDS [31,32]. NETs have multiple components, and the roles of each component participating in the pathogenesis of ALI/ARDS are still uncertain and deserve further investigation, as they could provide more direct information to optimize the clinical treatment.

In addition to suicidal NETosis, neutrophils can release NETs by releasing vesicles, and such neutrophils still have the ability to crawl, undergo chemotaxis, phagocytose and produce peroxides to kill pathogenic microorganisms [33]. The neutrophils from ARDS patients showed reduced apoptosis and an enhanced ability to produce NETs [34]. Vesicular secretion of NETs does not require the self-sacrifice of neutrophils, and it provides the opportunity for the transmembrane transport of NETs into the cytoplasm. In the present study, we found that NETs were involved in inflammatory pulmonary injury by activating the cytoplasmic *cGAS-STING* pathway. The *cGAS-STING* pathway can be activated by various DNA stimulations in the cytoplasm and promote the production of type I IFNs and various inflammatory factors [17]. NETs-DNA might be a powerful stimulatory signal for the cytosolic *cGAS-STING* pathway. However, as macromolecules in the extracellular matrix, how NETs activate the signal pathway located in the cytoplasm of airway epithelium cells is still unclear. It might be associated with special transmembrane transport, which deserves further exploration. Moreover, we also found that exogenous NETs intervention in vitro and in vivo resulted in DNA damage in airway epithelium cells. DNA damage can lead to DNA leakage, and the accumulation of DNA activates the *cGAS-STING* pathway in the cytoplasm. Recently, researchers found that the release of self-DNA activated the cytosolic *cGAS-STING* pathway and led to inflammatory injury, participating in the pathogenesis of pulmonary diseases such as allergic asthma and COPD [19,35]. Thus, self-DNA accumulation in the cytoplasm might be the reason for the activation of *cGAS-STING* in LPS-induced ALI. Interestingly, elevation in mitochondrial DNA (mtDNA) in the cytoplasm induced by mitochondrial fission could also activate the *cGAS-STING* pathway, which is an important cause of necroptosis in alveolar epithelial cells [36]. Several studies suggested that necroptosis is the critical form of cell death in LPS-induced acute lung injury [37,38]. Thus, we speculate that cytoplasmic mtDNA and damaged self-DNA can activate the *cGAS-STING* pathway, resulting in epithelial cell necroptosis, which needs to be further explored through more experiments. In summary, the signal transmission process from NETs to *cGAS-STING* is still unknown, and more investigations are required to elucidate it.

In this study, we uncovered the over-activation of the *cGAS-STING* pathway in LPS-induced ALI, while administration of H-151 successfully alleviated inflammatory lung injury. H-151 is a small-molecule inhibitor that interferes with *cGAS-STING* signaling by inhibiting *STING* palmitoylation. In this study, the evaluation of pulmonary tissue was based on various parameters such as pathological injury, inflammation, alveolar exudation, destruction of intercellular junctions and DNA damage in epithelial cells. It is possible that H-151 could rescue epithelial cells. Thus, the targeted inhibition of the *cGAS-STING* pathway is a valuable idea, which might be beneficial for optimizing the current treatment of ARDS/ALI. We pre-treated *mice* with H-151 before the establishment of ALI model according to the directions suggested in the literature. In this study, we paid more attention on the mechanism of acute lung injury, especially the upstream and downstream relationships between *cGAS-STING* pathway and NETs. Pretreatment of *mice* with the inhibitor did not affect cellular infiltration or MPO/NE levels in BALF, which suggested H-151 did not affect production and degradation of NETs. In addition, we found that DNase I reversed over-activation of *cGAS-STING* in acute lung injury. The above data suggested that *cGAS-STING* is located downstream of NETs. *cGAS-STING* pathway activation suppresses endothelial proliferation and vascular repair in inflammatory lung injury [39]. In this study, researchers extracted and synthesized a variety of small-molecule compounds that can regulate inflammatory response processes by interfering with the *cGAS-STING* pathway. Such molecules could blockade the *cGAS-STING* pathway by inhibiting DNA binding to *cGAS*, regulating the post-transcriptional modification of *cGAS*, inhibiting the palmitoylation of *STING*, interfering with the binding of *STING* to cGAMP and regulating the post-transcriptional modification of *STING* [40,41,42,43,44,45,46,47,48,49]. As most scientific achievements are still at the stage of basic study, more studies are needed, so that the theory can be transformed into clinical application.

There were some limitations in our study. Firstly, DNase I has broad activity, which could degrade various forms of DNA. As DNase I does not specifically target NETs, the effect of DNase I might be independent of NETs degradation. We found that DNase I could reduce *cGAS-STING* in vivo; however, such effects might also be independent of NETs degradation. Secondly, we used immunohistochemical staining of MPO and NE in pulmonary tissue to identify NETs in H-151 and DNase I intervention experiments. Both NE and MPO are critical components of NETs, and they showed consistent change trends after the same intervention in this study. However, neither NE nor MPO is a specific component of NETs. Possibly, the expression of NETs does not succumb to the entire quantification of NE or MPO. We will continue to investigate the study deeply in the future, and we intend to co-localize various components of NETs using a confocal microscope.

In the present study, we found that *cGAS-STING* regulated NETs-mediated airway inflammatory injury. In subsequent investigations, we will utilize *mice* with airway-specific *STING* knockout to validate the above conclusions and explore the related mechanism deeply. We validated the ALI *murine* model via the tracheal instillation of *P. aeruginosa*-derived LPS, which is a disease model related to intra-pulmonary infection. ARDS is a syndrome with multiple etiologies, and the pathophysiological process is not exactly the same in different etiologies. It is still unknown whether the *cGAS-STING* pathway serves as a common pathway for ARDS caused by different etiologies or just an effector pathway in a single category of ARDS. The heterogeneity of patients with ARDS should not be ignored. Clarification of the association between the mechanism and category of ARDS will help to identify the targeted population. Moreover, we also intend to collect clinical samples and clinical data of ARDS patients to further validate the role of NETs and the *cGAS-STING* pathway in ARDS.

## 4. Materials and Methods

### 4.1. Mice

Male C57BL/6 *mice* (wild-type, 6–8 weeks) were purchased from Shanghai Slack Laboratory Animal Company. The *mice* were housed in ventilated cages maintained in a clean environment with a constant temperature (25 ± 1 °C), humidity (60 ± 5%), 12 h light/dark cycle and free access to food and water. All the animal experiments were approved by the Ethics Committee of the Second Affiliated Hospital, Zhejiang University School of Medicine (No. 2021.126). Lipopolysaccharide (LPS) was an important component for the cell wall of Gram-negative bacteria. The LPS-induced acute lung injury *murine* model was quite mature and stable. It was used in numerous high-quality studies investigating acute lung injury [50,51]. In our study, *mice* were anesthetized with 1.25% Tribromoethanol (0.08 mg/kg) and administered intratracheal instillation of lipopolysaccharide (LPS, 10 mg/kg, Sigma-Aldrich, L9143) to construct the LPS-induced ALI model.

### 4.2. Experimental Design

Firstly, we compared the expression of NETs and *cGAS-STING* in BALF and pulmonary tissue between the ALI model (n = 6) and control (n = 6). Secondly, *mice* were randomly divided into four groups such as SHAM, ALI, SHAM/DNase I and ALI/DNase I, and each group contained 6 *mice*. *Mice* were administered an intravenous injection of deoxyribonuclease I (DNase I, 20 mg/kg, Sigma-Aldrich, St. Louis, MI, USA, 10104159001) to inhibit NETs, which was performed 1h after model construction. Thirdly, another 24 *mice* were randomly divided into four groups including SHAM, ALI, SHAM/H-151 and ALI/H-151, and each group contained 6 *mice*. H-151 is a potent and selective antagonist of *human STING* (h*STING*) and *murine STING* (m*STING*) [40]. These *mice* received an intraperitoneal injection of H-151 (30 mg/kg, MCE, HY-112693) 30 min before ALI model construction. We extracted exogenous NETs from *murine* neutrophils (isolated from bone marrow) as well as *human* neutrophils (differentiated from HL-60). *Mice* were divided into the following groups: NETs (n = 6), NETs + H-151 (n = 6) and SHAM (n = 6). *Mice* that received intratracheal instillation of *murine* NETs (mNETs) were regarded as the model group (NETs group), whereas the SHAM group was the blank control. *Mice* in the NETs + H-151 group were administered an intraperitoneal injection of H-151 to inhibit the *cGAS-STING* pathway, which was performed 30 min prior to intratracheal instillation of NETs. *Mice* were sacrificed 24 h after model establishment. Lung tissue, serum, plasma and bronchoalveolar lavage fluid (BALF) were harvested and stored. The procedure was performed in accordance with the National Research Council’s Guide for the Care and Use of Laboratory Animals.

### 4.3. Cell Counts in BALF

Twenty-four hours after the intratracheal instillation of LPS, the *mice* were sacrificed. The trachea was flushed with ice-cold PBS 3 times to obtain BAL cells. Equal volumes of BALF and 0.2% glacial acetic acid were mixed. A micro-volume of the above mixture was added to the blood cell counting plate, and total cell counts were performed under a microscope. BALF was centrifuged at 6000 rpm for 10 min at 4 °C to acquire the supernatant and cell pellets. Cell pellets were resuspended with PBS, after which a cell smear was performed. Slides were stained with Giemsa–Wright dye and were then observed under a microscope. Cell counts were performed in a single-blind fashion and were conducted by one investigator independently.

### 4.4. Wet/Dry Ratio Determination

The obtained fresh right lungs were weighed, and then the lung samples were baked in a 68 °C oven. The samples were weighed and recorded every 24 h until the weight of the lung tissue no longer changed. The first and last weights were regarded as the wet and dry weights, respectively, based on which the wet/dry ratio was calculated.

### 4.5. Isolation of Murine Neutrophils

*Mice* were sacrificed, and then femurs and tibias were collected in sterile culture dishes. Bone marrow cell suspensions were obtained by performing incisions of bone epiphyses. The marrow cavity was flushed with complete culture medium (RPMI 1640 medium with 10% FBS, 1% penicillin-streptomycin and 2 mM EDTA) and, subsequently, filtered through a sterile 100 μm nylon mesh cell strainer. Then, 100% Percoll was prepared by mixing Percoll (Sigma-Aldrich, St. Louis, MI, USA, P1644) and 10X PBS, the volume ratio of which was 9:1. After its preparation, 100% Percoll was diluted with 1X PBS to acquire gradient Percoll separation solutions. Subsequently, 72%, 64% and 54% Percoll separation solutions (2 mL per gradient) were added to the centrifuge tube layer by layer, and marrow cells were laid carefully on the uppermost layer. The tube containing Percoll and marrow cells was centrifuged at 900× *g* for 30 min at room temperature (accelerate rate 1, decelerate rate 1, ThermoFisher). The cells suspended between 64% Percoll and 72% Percoll were *murine* neutrophils.

### 4.6. Induction of Human Neutrophils

HL-60 cells (*human* promyelocytic leukemia cells) were transformed cells purchased from Shanghai EK-Bioscience Biotechnology Co., Ltd (Shanghai, China) and could be induced to differentiate into *human* neutrophils. HL-60 cells were cultured in IMDM medium (Biosharp, Guangzhou, China, BLA312A) containing 20% FBS, 2 mM L-glutamine and 1% penicillin-streptomycin. HL-60 cells in the logarithmic growth phase were further suspended in IMDM medium containing 1.25% DMSO and incubated for 3–4 days. HL-60 differentiation was evaluated via flow cytometry analysis with CD11b^+^ expression, a critical marker for neutrophils. Mature neutrophil differentiation reaching 80% met the requirements and could be used for the next experiment.

### 4.7. Induction and Isolation of Human NETs and Murine NETs

Neutrophils were suspended with RPMI 1640 medium supplemented with 10% FBS (Noverse, Hangzhou, China, NFBS-2500A), 1% penicillin-streptomycin (Gibco, Grand Island, NY, USA, 15140122). Then, 3 × 10^7^ neutrophils (1 × 10^7^ cells/mL) were seeded per well in a 6-well-plate, stimulated with PMA (Sigma-Aldrich, St. Louis, MI, USA, P8139) and incubated for 4 h at 37 °C, 5% CO_2_. After incubation, the medium was removed carefully without disturbing the NETs on the well bottom. Released NETs and cell debris were collected with 500 ul pre-cooled Hank’s Balanced Salt Solution (Beyotime, Jiangsu, China, C0218). Cell debris and NET fragments were separated by centrifugation at 4 °C, 400 g, for 5 min. The supernatant enriched in NETs was transferred to a fresh tube and frozen at −80 °C [52].

### 4.8. Enzyme-Linked Immunosorbent Assays

NETs in BALF were detected as MPO-DNA and NE-DNA by ELISA. Capture antibodies (anti-MPO or anti-NE, 1:2000 dilution in sterile PBS) were applied to 96-well high-binding-capacity ELISA microplates. The ELISA plates were incubated overnight at 4 °C, followed by three repeated washes with PBS-Tween 20. Then, the ELISA plates were blocked with 5% BSA (200 μL/well) and incubated for 2h at room temperature. Then, the plates were repeatedly washed again with PBS-Tween 20 (200 μL/well). BALF that was collected from the ALI model and control was added to the ELISA plates. Then, the ELISA plates were incubated with samples overnight at 4 °C to allow the protein component of the NETs to bind to capture antibodies. The plates were repeatedly washed again with PBS-Tween20. *Murine* HRP-labeled anti-dsDNA antibody (Cell Death Detection ELISA, Roche, Switzerland,1:500 dilution) was added to the plates, which were then incubated for 1h in the dark at room temperature. Then, the plates were repeatedly washed again with PBS-Tween 20. TMB peroxidase substrate was applied to each well for 30 min (50 μL/well), and 1 M HCl was added to each well to stop the reaction (50 μL/well). Absorbance was read at 405 nm using a microplate photometer. NE and MPO in *murine* BALF were detected using commercial kits for the detection of *murine* Myeloperoxidase (R&D Systems, USA, DY3667) and Neutrophil Elastase (R&D Systems, USA, DY4517-05), following the manufacturers’ instructions. RAGE and SP-D in *murine* serum were detected using commercial kits for the detection of *murine* RAGE (R&D Systems, USA, MRG00) and *murine* SP-D (R&D Systems, USA, MSFPD0), following the manufacturer’s instructions.

### 4.9. Real-Time Quantitative Polymerase Chain Reaction (RT-qPCR)

Total RNA was extracted using Trizol reagent (Invitrogen, Carlsbad, CA, USA, 15596018), and cDNA was synthesized using PrimeScript^TM^ RT Reagent Kit (Takara, Japan, RR037A) according to the manufacturer’s recommendations with TB Green Premix Ex Taq (Takara, Japan, RR420A). Real-time PCR data were analyzed using the 2^−ΔΔCt^ method, and gene expression was normalized to GAPDH. Primers used in this study were synthesized by Tsingke Biotechnology Co., Ltd. (Beijing, China), and the sequences are shown in Supporting Information (Table S1).

### 4.10. RNA-Seq Data Analysis

A transcriptome library was constructed based on mRNA obtained from pulmonary tissue of the ALI *murine* model and control. Raw reads were obtained by performing sequencing using the BGISEQ platform, from which clean reads were obtained by filtering out the reads with a low quality, adapter contamination and an unknown base N content. Then, clean reads were aligned to the reference genome sequence (GCF_000001635.26_GRCm38.p6) in the National Center for Biotechnology Information (NCBI) database using the Bowtie2 software. Differentially expressed genes (DEGs) were identified using the DESeq2 package in R (4.2.0 version) based on a Bonferroni-adjusted *p*-value < 0.01 and a |log2 foldchange| > 2. Kyoto Encyclopedia of Genes and Genomes (KEGG) enrichment analysis was performed using the ClusterProfiler package in R, and up-regulated pathways in ALI (*p*-value < 0.05) were visualized using a bubble chart. Gene set enrichment analysis (GSEA) was performed in R following the standard procedure [53]. “|Normalized enrichment score (NES)|>2” and “*p*-value < 0.05 and adjust q-value < 0.25” were used as the judgment thresholds to determine significant differences between groups in GSEA. Gene set variation analysis (GSVA) was conducted in R to analyze the difference between ALI and the control based on the gene set, and “adjust *p* < 0.05” was used as the cutoff criterion for significance. A GSEA map, heatmap and bubble map were constructed using the gseaplot2 package, enrichplot package, ggplot 2 package and pheatmap package in R.

### 4.11. Small Interfering RNA

BEAS-2B cells were plated in 6-well plates and cultured for 24h before transfection. siRNA (si-h-*STING* and si-negative-control, RiboBio, Guangzhou, China), OptiMEM^TM^ (ThermoFisher, Waltham, MA, USA, 31985062) and Lipofectamine™ RNAiMAX (ThermoFisher, Waltham, MA, USA, 13778150) were mixed to establish a transfection complex by following the manufacturers’ instructions. Then, BEAS-2B cells were cultured with RPIM 1640 (Gibco, C11875500BT) supplemented with the transfection complex for 24 h, and cells were collected for downstream experiments.

### 4.12. Lung Histology

The same lung lobes from *mice* in different groups were preserved in 4% paraformaldehyde for 24 h. Then, the lung tissue was embedded in paraffin wax, sliced and stained with hematoxylin-eosin. The thickness of the tissue sections was 3–4 um. Lung histology sections were observed via microscope, and evaluation of ALI was performed by a single researcher independently. Alveolar edema, pulmonary hemorrhage, leukocyte infiltration and alveolar septal thickening were evaluated, and each item had four ranks, namely normal (score of 0), mild (score of 1), moderate (score of 2) and severe (score of 3), based on which the assessment of ALI was calculated.

### 4.13. Immunohistochemistry

Lung tissue slices were blocked with 5% BSA (Beyotime, Jiangsu, China, ST2254) following microwave antigen retrieval in citric acid solution (Hangzhou Hulk Biotechnology Co., Ltd., Hangzhou, China, HK1222) for 30 min. Slices were incubated with primary antibodies at 4 °C overnight, and secondary antibodies were added after three consecutive washes with PBS. The following antibodies were used: anti-*cGAS* (Proteintech, Wuhan, China, 26416-1-AP, 1:500), anti-*STING* (Proteintech, Wuhan, China, 19851-1-AP, 1:2000), anti-MPO (Abcam, Cambridge, UK, ab45977, 1:100), anti-NE (Abcam, Cambridge, UK, ab68672, 1:200) and anti-γH2AX (Abcam, Cambridge, UK, an81299, 1:200). DAB dye was added to show the positive expression, whereas 4′,6-diamidino-2-phenylindole (DAPI) was added to perform nuclear staining. Images were captured by a microscope (Leica, Wetzlar, Germany, DM2500).

### 4.14. Immunofluorescence Staining of Cells and Lung Tissue

Cells were blocked with 4% paraformaldehyde and permeabilized with 0.5% Triton X-100 in PBS for 20 min, whereas lung samples were blocked with 3% BSA following microwave antigen retrieval. Samples were incubated with primary antibodies at 4 °C overnight, and the following antibodies were used: anti-histone (Abcam, Cambridge, UK, ab1220, 1:500), anti-γH2AX (Abcam, Cambridge, UK, ab81299, 1:500), anti-*STING* (Proteintech, Wuhan, China, 66680-1-lg, 1:1000), anti-E-cadherin (Proteintech, Wuhan, China, 20874-1AP, 1:1000). After three consecutive washes with PBS, secondary antibodies of the corresponding species were added and incubated for 50 min at room temperature protected from light. TSA reagent was added to amplify the red fluorescence signal, and then antigen retrieval and peroxidase blocking were performed again after washing for three times. Another primary antibody was incubated with the samples after blocking, and the following antibodies were used: anti-ZO-1 tight junction (Abcam, Cambridge, UK, ab221547, 1:400), anti-GM130 (Invitrogen, Carlsbad, CA, USA, MA5-35107, 1:200), anti-Calnexin (Abcam, Cambridge, UK, ab75801, 1:100), anti-MPO (Abcam, Cambridge, UK, ab45977, 1:500), anti-SFTPC (Abcam, Cambridge, UK, ab211326, 1:500), anti-NE (Abcam, Cambridge, UK, ab68672,1:500). The TSA step was repeated to amplify the green fluorescence signal after adequate washing. Then, secondary antibodies of the corresponding species were added and incubated for 50 min. The secondary antibodies included Alexa Fluor 488-labeled goat anti-rabbit IgG (Beyotime, Jiangsu, China), Alexa Fluor 647-labeled goat anti-mouse IgG (Beyotime, Jiangsu, China) and Alexa Fluor 647-labeled goat anti-rabbit IgG (Beyotime, Jiangsu, China). Nuclear staining was performed with 4′,6-diamidino-2-phenylindole (DAPI). Images were captured by a Pannoramic scan II (3D HISTECH) and fluorescence microscope (LAS X software, Leica, Wetzlar, Germany).

### 4.15. Statistical Analysis

Data are described as the mean ± standard deviation (SD) and were analyzed using GraphPad Prim 8. For comparisons between two datasets, Student’s t test was applied if they were distributed normally; otherwise, the Mann–Whitney test was applied. For more than two groups, one-way ANOVA was applied, and Tukey analysis was used as a post hoc test. A value of *p* < 0.05 was considered statistically significant. Asterisks in the figures represent the following: * *p* < 0.05; ** *p* < 0.01; *** *p* < 0.001. Values are from ≥3 separate experiments.

## 5. Conclusions

In this study, we found overexpression of NETs and over-activation of the *cGAS-STING* pathway in LPS-induced ALI. This is the first study revealing that NETs and *cGAS-STING* are involved in the pathogenesis of acute lung injury. Moreover, we also found that the *cGAS-STING* pathway regulated NETs-mediated inflammatory lung injury in LPS-induced ALI. Therefore, overexpressed NETs components and *cGAS-STING* are novel potential biological markers and therapeutic targets for ARDS/ALI.

## Data Availability

The data presented in this study are available in the article.

## Supplementary Materials

The following supporting information can be downloaded at: https://www.mdpi.com/article/10.3390/ijms24065125/s1.
