# Supplementary material for: NETs Promote Inflammatory Injury by Activating cGAS-STING Pathway in Acute Lung Injury
Source: Int J Mol Sci. 2023 Mar 7;24(6):5125. doi: 10.3390/ijms24065125 (PMC10049640; doi:10.3390/ijms24065125)
Supplement: Supplementary file 1 [file ijms-24-05125-s001.zip › ijms-2144126-supplementary.pdf]

Supplemental Figure S1: Successful establishment of acute lung injury model.

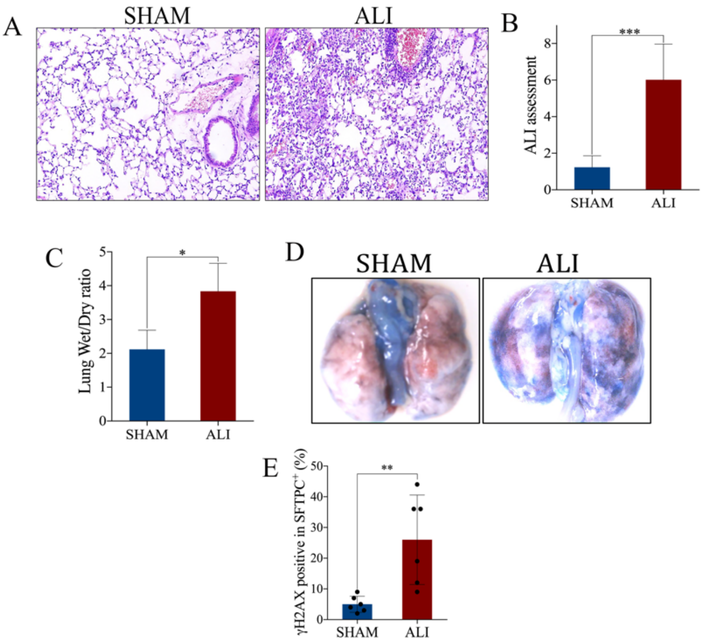

Legends: Healthy C57BL/6 mice were intratracheally administered LPS (10 mg/kg) or SHAM (PBS). (A) Representative image of H&E stain of lung tissue 24 h after LPS or PBS administration (X100). (B) ALI assessment score. (C) Lung wet weight to dry ratio. (D) Representative images of Evans blue. (E) The percentage of γH2AX positive cells in SFTPC+ in lung tissue with immunofluorescence co-localization. Data were presented as Mean ± SD, *p<0.05, **p<0.01, ***p<0.001. Comparison between two groups were analyzed by an unpaired t test.

Supplemental Table S1. Sequences of the primers used to quantitate gene expression.

Gene	Species	Primer	Sequence (5'-3')
GAPDH	Human	Forward	CCACGATAACACCAGCTTCG
GAPDH	Human	Reverse	ACTTGAGCATGTAGGCCTGT
cGAS	Human	Forward	GAAGAAACATGGCGGCTATC
cGAS	Human	Reverse	TGAGGGTTCTGGGTACATACG
STING	Human	Forward	CAGGCACTGAACATCCTCCT
STING	Human	Reverse	ATATACAGCCGCTGGCTCAC
GAPDH	Mouse	Forward	TCGAGTCGCGTCCACC
GAPDH	Mouse	Reverse	GGGAGCATCGTCGCCC
cGAS	Mouse	Forward	CCGAAGAGCGGCAGATATGGAA
cGAS	Mouse	Reverse	CGTGGCTTCTCCGTGGTGTCC
STING	Mouse	Forward	AAATAACTGCCGCCTCATTG
STING	Mouse	Reverse	TGGGAGAGGCTGATCCATAC
